# Chimeric antigen receptor T‐cell therapy in diffuse large B‐cell lymphoma: Evaluating axicabtagene ciloleucel in the ZUMA‐7 trial

**DOI:** 10.1002/hem3.70119

**Published:** 2025-03-24

**Authors:** Vadim Lesan, Cristian Munteanu

**Affiliations:** ^1^ Hematology and Oncology Department Saarland University Hospital Homburg Germany; ^2^ Statistician Saarland University Hospital Homburg Germany

Chimeric antigen T‐cell (CAR‐T) therapy is the new standard of therapy in patients with refractory or early relapsed diffuse large B‐cell lymphoma (r/r DLBCL).^1^ Axicabtagene ciloleucel (Axi‐cel) was one of the first therapies to show superior event free survival (primary endpoint of the study) and significant overall survival (OS) benefit in this context.^2^ Recently published data on subsequent anti‐lymphoma therapies after second‐line axicabtagene ciloleucel (Axi‐Cel) imply that outcomes are better when Axicel is given in earlier lines of therapies.^3^ Further, reanalysis of the ZUMA‐7 trial outcome data regarding the metabolic tumor volume (MTV) revealed that Axicel improved event‐free survival and progression‐free survival over the standard of care irrespective of MTV.^4^ Although we acknowledge the positive impact of Axicel in the treatment of patients with DLBCL, we raise some concerns about moving Axicel further in the frontline therapies.

Analyzing the outcome results for subsequent therapies after Axi‐cel and SOC in the context of the previous reports from the ZUMA‐7 trial, we observed some important discrepancies. First, the median time to third‐line therapy was lower in the SOC arm compared to the Axi‐Cel arm (2.8 vs. 4.4 months). This difference could be either due to higher proportion of aggressive disease and/or poor prognostic factors in SOC arm or due to greater investigator's willingness to declare refractory and/or progressive disease in the SOC arm compared to Axi‐Cel arm. Although, the absence of bridging therapy has precluded the enrollment of patients with aggressive disease in the initial report of the ZUMA‐7 trial, only two patients had progressive disease before Axi‐Cel infusion. At a median of 29 days from leukapheresis to infusion without bridging therapy, this results in a progression rate of 0.06 patients per day. On the other side, seventy patients progressed in the SOC arm before autologous stem cell transplantation (ASCT). Since data on the duration between the start of the salvage chemotherapy and ASCT was not reported, estimating it at about 60 days (corresponding to two cycles of salvage chemotherapy at a duration of 21 days per cycle and adding time for leukapheresis and pre‐ASCT screening) will result in a progression rate of 1.1 patients per day. Altogether, these results point to a higher proportion of aggressive disease in the SOC arm and potential survivorship bias in the Axi‐Cel arm. The role of investigator's willingness to declare more refractory and/or progressive disease in the SOC arm should be minimal, since the responses in the initial report were assessed per‐blinded central review. Still, ZUMA‐7 is an open‐label study, and assessment bias might have occurred. Blinded outcome assessment alone is not sufficient to overcome the information bias upon which the response assessment is based.^5^

Second, looking at the efficacy of the third‐line therapy in the SOC arm, patients receiving cellular therapies (autologous or allogeneic CAR‐T therapies) had better median progression‐free survival and overall survival than patients receiving other non‐cellular therapies. Still, achieving a median OS of 9.5 months (95%CI: 6.6‐15.4 months) with non‐cellular therapies in third‐line LBCL after salvage therapy, seems inappropriately long. Putting it into perspective, the median OS in the patients failing to undergo ASCT in the CORAL study, was only 4.4 months.^6^ The pooled data of the SCHOLAR‐1 study showed a median OS of only 6.3 months in patients with refractory or relapsed (≤12 months) DLBCL failing first‐line chemotherapy.^7^ As such, the unusually long overall survival after third‐line therapy in the ZUMA‐7 trial could point to an undertreatment in the second therapy line. Indeed, the median number of chemotherapy cycles in the SOC arm of ZUMA‐7 was reported at 2, whereby per protocol patients could have received either two or three cycles of salvage chemotherapy.^1^

Third, the median OS in the patients receiving third‐line cellular immunotherapy after SOC is shorter compared to the ZUMA‐1 trial (16.3 vs. 25.8 months), even though the follow‐up time and patient characteristics were very similar.^8^ This points again to a more difficult to treat patient population in the SOC arm. Interestingly, the median metabolic volume (MTV), an important prognostic factor, was higher in the ZUMA‐7 trial compared to the ZUMA‐1 trial (231.07 vs. 147.5 mL).^4^, ^9^ Although the investigators report similar median MTV in both SOC and Axi‐Cel arms of the ZUMA‐7 trial, one can argue that individual MTV values could be higher in SOC and still result in insignificant median difference between SOC and Axi‐Cel arm (Figure 1). In support of this hypothesis, investigators report, based on log‐rank statistics for the primary endpoint, a significantly lower MTV volume threshold in Axi‐Cel arm compared to SOC arm (57.68 vs. 267.88 mL). ^4^

**Figure 1:**
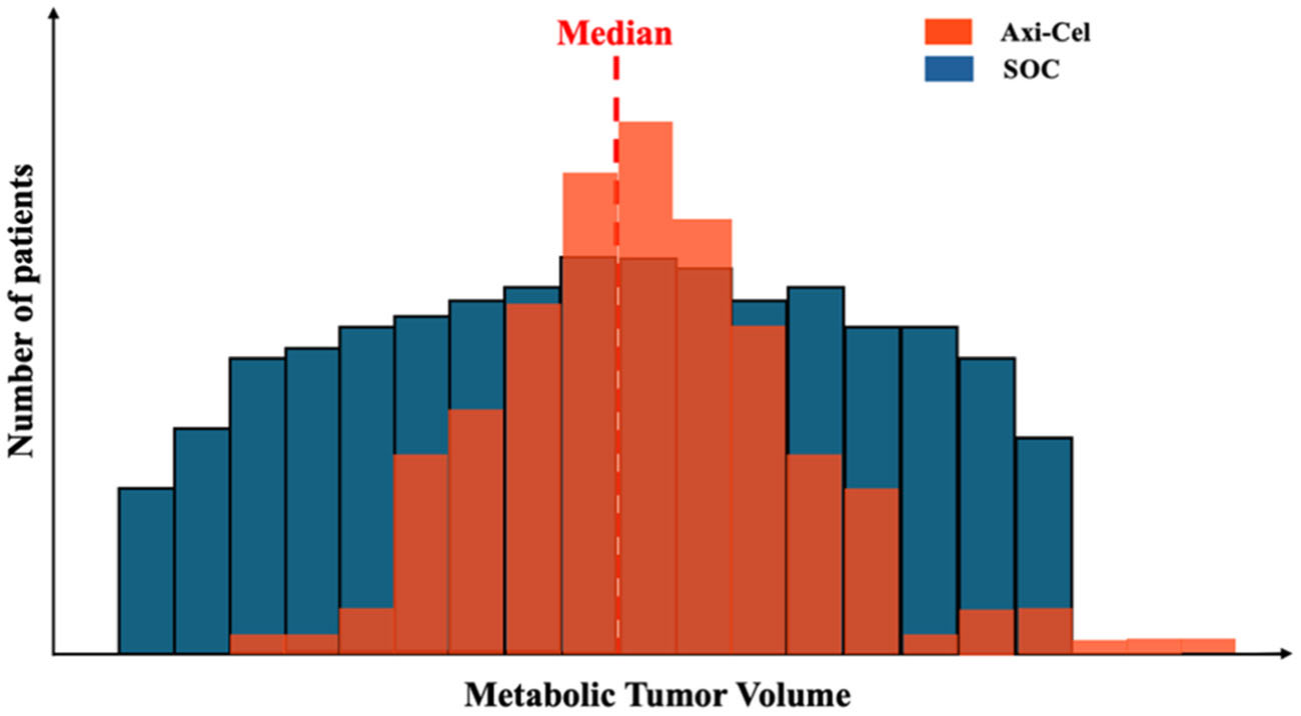
**Schematic representation of equal median but different individual distribution of MTV in Axi‐Cel and SOC arms**.

Finally, the ZUMA‐7 trial was not testing SOC therapy versus Axi‐Cel in a sequential manner. If this would have been the case, all patients relapsing after SOC should have received Axi‐Cel as the new standard for third‐line therapy in DLBCL. As reported, only half of patients relapsing after SOC received subsequent autologous anti‐CD‐19 CAR‐T cell therapy. This unplanned crossover is also debatable, at least from an ethical point of view.^3^ One can make the definitive argument about the OS benefit of Axi‐cel in the second versus third therapy line only with crossover rates reaching nearly 100%.

In conclusion, patients in the SOC arm of the ZUMA‐7 trial had higher rates of progression under therapy, could have been undertreated both on trial therapy and at progression, and had probably higher tumor burden. All these factors may contribute to an overestimation of Axi‐Cel's effectiveness, potentially influencing clinical decision‐making for both practitioners and their patients. Figure 2 summarizes how the OS benefit in favor of Axi‐Cel could have been overestimated in the ZUMA‐7 trial.

**Figure 2:**
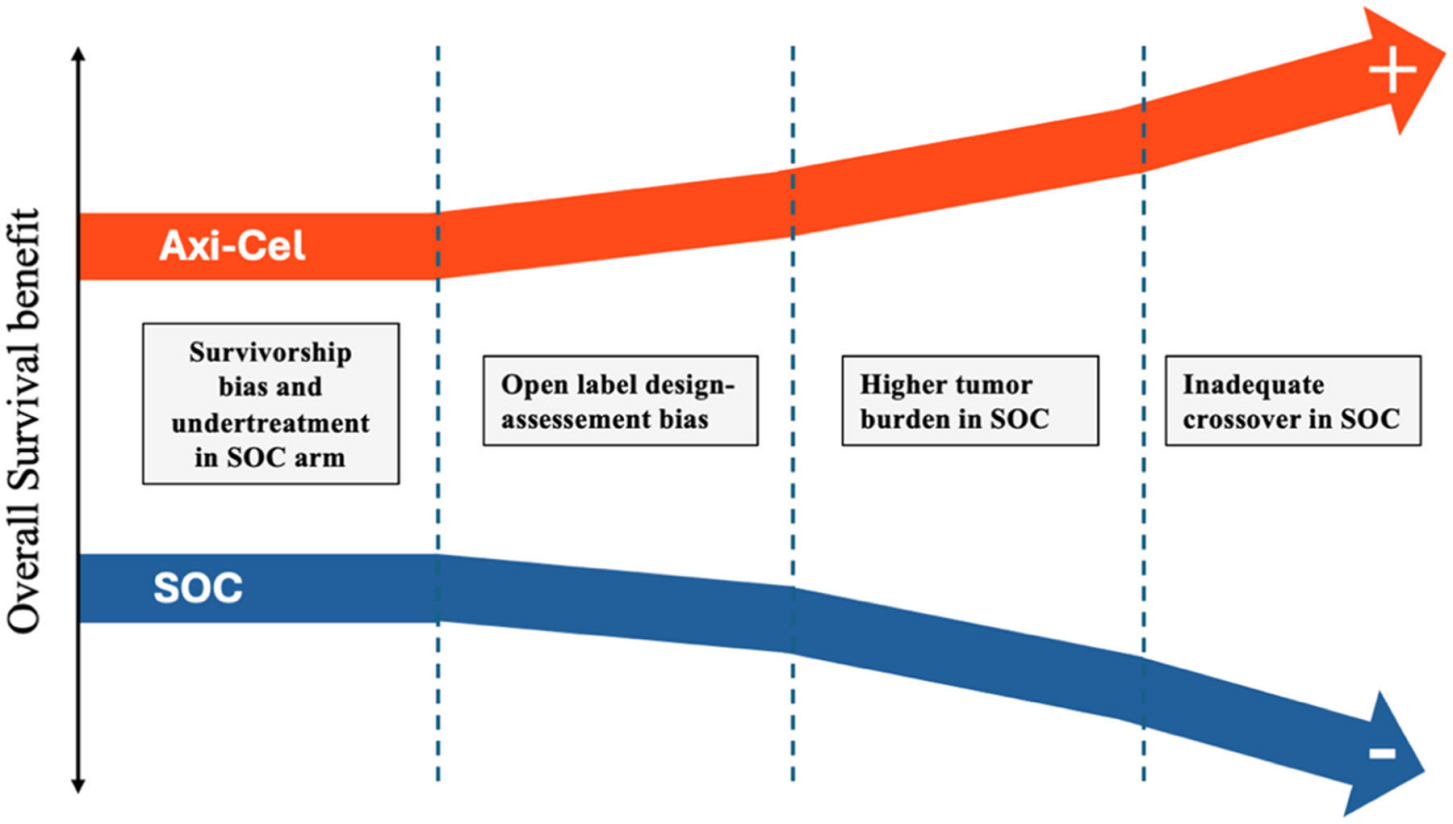
Summary of possible factors overestimating OS benefit in Axi‐Cel arm.

## AUTHOR CONTRIBUTIONS

**Vadim Lesan**: conceptualization; writing—original draft. **Cristian Munteanu**: writing—review and editing.

## CONFLICT OF INTEREST STATEMENT

Vadim Lesam has received honoraria from AbbVie; and travel grants from AbbVie, Janssen, Pfizer, and Pierre Fabre. Cristian Munteanu does not have any conflicts of interest.

## ETHICS STATEMENT

Not applicable.

## FUNDING

No funding was provided.

## Data Availability

Not applicable.
